# Altering Automatic Verbal Processes with Transcranial Direct Current Stimulation

**DOI:** 10.3389/fpsyt.2012.00073

**Published:** 2012-08-06

**Authors:** Tracy D. Vannorsdall, David J. Schretlen, Megan Andrejczuk, Kerry Ledoux, Laura V. Bosley, Jacqueline R. Weaver, Richard L. Skolasky, Barry Gordon

**Affiliations:** ^1^Department of Psychiatry and Behavioral Sciences, Johns Hopkins University School of MedicineBaltimore, MD, USA; ^2^Russell H. Morgan Department of Radiology and Radiological Sciences, Johns Hopkins University School of MedicineBaltimore, MD, USA; ^3^Department of Neurology, Johns Hopkins University School of MedicineBaltimore, MD, USA; ^4^Department of Orthopaedic Surgery, Johns Hopkins University School of MedicineBaltimore, MD, USA; ^5^Cognitive Science Department, Johns Hopkins University School of MedicineBaltimore, MD, USA

**Keywords:** verbal fluency, clustering, switching, transcranial direct current stimulation

## Abstract

**Background:** Word retrieval during verbal fluency tasks invokes both automatic and controlled cognitive processes. A distinction has been made between the generation of words clusters and switches between such clusters on verbal fluency tasks. Clusters, defined by the reporting of contiguous words that constitute semantic or phonemic subcategories, are thought to reflect relatively automatic processing. In contrast, switching from one subcategory to another is thought to require a more controlled, effortful form of cognitive processing. **Objective:** In this single-blind, sham-controlled experiment, we investigated whether anodal and cathodal transcranial direct current stimulation (tDCS) can differentially modify controlled or automatic processes that support lexical retrieval, as assessed by clustering and switching on verbal fluency tasks, in 24 healthy right-handed adults. **Methods:** Participants were randomly assigned to receive 1 mA of either anodal (excitatory) or cathodal (inhibitory) active tDCS over the left dorsolateral prefrontal cortex in addition to sham stimulation over the same region in counterbalanced order. Participants engaged in various cognitive activities during the first 23 min of stimulation. Then, during the final segment of each 30-min session, they completed letter- and category-cued word fluency tasks. **Results:** Participants reported more words on category-cued word fluency tasks during anodal than sham stimulation (25.9 vs. 23.0 words; *p* = 0.055). They also showed a net increase in the number of clustered words during anodal stimulation compared to a net decrease during cathodal stimulation (1.3 vs. −1.5 words; *p* = 0.038). **Conclusion:** tDCS can selectively alter automatic aspects of speeded lexical retrieval in a polarity-dependent fashion during a category-guided fluency task.

## Introduction

Overt behaviors are often generated by a variable admixture of automatic and controlled processes. Verbal fluency tasks have been widely used to assess these processes governing lexical retrieval in healthy adults and various patient populations for both research and clinical purposes. During verbal fluency tasks, it has been hypothesized that internal or external cues activate chains of automatic associations, resulting in the successive generation of related words (i.e., “clustering,” as in the contiguous generation of the words shirt, socks, skirt, and shoes on a letter-cued fluency task using the letter “s”). When these automatic associations dissipate, then effortful cognitive control processes are used to find new cues, thereby initiating another automatic chain (i.e., “switching,” as is seen when one goes from providing exemplars of farm animals to providing exemplars of zoo animals on an category-cued fluency task with the category “animals”). The operations of these two distinct processes, automatic and controlled, are thought to be reflected in the nature of the items produced, and in the time of production: automatic processes give rise to clusters of related items with relatively short inter-item intervals, while controlled processes lead to switches among subcategories after longer intervals.

For verbal fluency tasks, there is evidence that automatic processes are associated with the dominant (left) posterior temporal-parietal regions, while controlled processes are associated with the dominant (left) prefrontal region (Hirshorn and Thompson-Schill, 2006). Different verbal fluency tasks likely invoke varying combinations of automatic or controlled processes, and hence different weightings of anatomic dependence. Letter fluency tasks have been associated with greater activation of the left frontal lobe (as assessed by functional magnetic resonance imaging, fMRI), whereas category fluency tasks activate left temporal regions to a greater extent (Birn et al., 2010).

Clustering and switching processes are modulated by a number of participant characteristics. Evidence suggests that older healthy adults switch less frequently on category-cued fluency tasks and produce larger clusters on letter-cued fluency tasks than younger adults (Troyer et al., 1997). Alzheimer disease and focal lesions of the left temporal lobe are associated with the production of smaller clusters than are typically produced by healthy age-matched controls (Troyer et al., 1998). Conversely, patients with Parkinson’s disease and multiple sclerosis appear to switch less frequently than both healthy controls and some patient populations, although their clustering remains intact (Troster et al., 1998).

Transcranial direct current stimulation (tDCS) involves passing weak direct electrical current through the intact scalp to alter the functioning of underlying cerebral tissues. A rapidly growing body of evidences demonstrates that tDCS can induce changes in physical and cognitive functioning (Stagg and Nitsche, 2011). Stimulation with tDCS is thought to produce a relatively localized, polarity-dependent alteration of the electrical potential of the cortical tissue beneath the scalp electrode. The effects of these alterations can be excitatory with the application of anodal stimulation, or inhibitory with the application of cathodal stimulation. As tDCS is typically applied, 1–2 mA of direct current is administered via 25–35 cm^2^ saline-soaked sponges for up to 40 min. Under these conditions, the technique has been found to be safe and unobtrusive (Iyer et al., 2005). Depending on the duration of stimulation and the experimental situation, some effects of tDCS can persist for minutes, hours, days, or even more than a week (Reis et al., 2009). The ability of tDCS to activate or inhibit brain function over short and long time intervals and the fact that active stimulation can be counterbalanced with sham stimulation make tDCS an attractive tool for investigating and perhaps enhancing cognitive processes.

Initial investigations of tDCS as a means of modifying cognitive functioning have shown some promise in improving implicit learning of a motor sequence, probabilistic learning, memory consolidation, and working memory, among other skills (Miniussi et al., 2008). A few studies have found that anodal stimulation can improve selected aspects of language functioning in healthy adults. For example, tDCS has been shown to improve language learning (Floel et al., 2008) and facilitate implicit learning of an artificial grammar in healthy adults (de Vries et al., 2010). Further, anodal tDCS applied to the left frontal lobe has been found to shorten picture naming latencies among healthy adults in several studies (e.g., Sparing et al., 2008). A fMRI study found that decreased naming latencies following tDCS were associated with decreased blood oxygen level-dependent signal in the left inferior frontal cortex (Holland et al., 2011). Some tDCS-related improvements in picture naming accuracy have also been documented in persons with post-stroke aphasia (e.g., Baker et al., 2010). On verbal fluency tasks, Iyer et al. (2005) found that anodal tDCS applied to the left prefrontal cortex produced modest, though significant, increases in the total number of words produced on a letter fluency task in healthy adults. Cattaneo et al. (2011) also found a facilitative effect of anodal tDCS relative to sham on overall productivity during letter- and category-cuedword fluency tasks during anodal stimulation of Broca’s area in healthy adults.

Here we sought to extend prior findings and further investigate the ability of tDCS to modify the automatic and controlled aspects of speeded verbal production among healthy adults. Based on prior neuroimaging and stimulation studies, we hypothesized that anodal and cathodal tDCS applied over the left prefrontal cortex would enhance and impede, respectively, verbal fluency production. We also hypothesized that stimulating the left prefrontal cortex would produce greater polarity-dependent effects on letter-cued than category-cued fluency, as well as greater effects on controlled (i.e., switching) than automatic (i.e., clustering) word retrieval processes.

## Materials and Methods

### Participants

Forty adults were recruited from the Johns Hopkins University via word-of-mouth and from the Baltimore metropolitan area using Craigslist. All participants were healthy, right handed (as assessed by the Edinburgh Inventory), native English speakers. All participants also completed at least 12 years of schooling (*M* = 14.6 years, SD = 2.3) and were of at least average estimated intelligence (*M* = 104.2, SD = 8.0) based on the Hopkins Adult Reading Test (HART; Schretlen et al., 2009). This study was approved by the Johns Hopkins Medicine Institutional Review Board, and all participants provided written informed consent.

During the random assignment of participants to the anodal/sham or cathodal/sham condition, younger and more highly educated adults were markedly over-represented in the anodal group. Consequently, we conducted a secondary series of exploratory analyses after better equating the two experimental conditions with respect to age and educational attainment. Specifically, we excluded subjects as necessary, starting with the youngest participants from the anodal/sham group and the least educated participants from the cathodal/sham group, in order to form two equal-sized groups that were matched for age, education, and estimated intelligence (all *p*s > 0.05). Through this process we retained a final sample of 24 adults, aged 24–55 years (*M* = 35.7; SD = 10.1). Characteristics of the final sample of study participants are shown in Table 1.

**Table 1:** **Characteristics of study participants by experimental condition**.

Characteristic	Experimental condition^1^		Statistic	*p*-Value
	Anodal (*N* = 12)	Cathodal (*N* = 12)		
Sex, male/female	6/6	5/7	X^2^_(1)_ = 0.17	0.68
Age^2^ (years)	37.9 ± 11.3	33.5 ± 8.7	*t*_(22)_ = 1.08	0.29
Education^2^ (years)	14.8 ± 2.0	15.3 ± 2.9	*t*_(22)_ = −0.41	0.69
Estimated IQ^2^	104.7 ± 7.5	103.5 ± 10.3	*t*_(22)_ = 0.33	0.74

*^1^Anodal = active anodal plus sham stimulation. Cathodal = active cathodal plus sham stimulation*.

*^2^Values expressed as mean ± standard deviation*.

### Procedures

In this single-blind experiment, subjects were assigned to receive one 30-min session of either anodal (facilitative) or cathodal (inhibitory) active tDCS together with 30 min of sham stimulation using a random number sequence. Active and sham stimulation were administered in counterbalanced order and separated by a 90-min washout period.

Stimulation was applied via a constant current stimulator (Iomed Phoresor II Model PM850) using two saline-soaked sponge electrodes (5.2 cm × 5.2 cm). The active electrode was placed over the left prefrontal region (F3 according to the 10-20 International EEG positioning system), and the reference electrode was placed over the vertex (Cz). In the active stimulation conditions, current was ramped up to 1.0 mA over 30 s and remained at 1 mA for the remainder of the 30-min session. Consistent with prior research, current in the sham stimulation condition was ramped up to 1.0 mA and then covertly ramped back down to 0 mA over 60 s, thereby habituating participants to the sensations (e.g., warmth, tingling) of tDCS (Nitsche et al., 2008).

Because maximal gains usually are achieved when tDCS is coupled with behavioral training (Reis et al., 2009), participants spent the first 24 min of each stimulation session engaging in expressive language tasks such as object naming and oral reading. The activities and stimuli were identical in the active and sham conditions. During the last 6 min of each 30-min session, participants completed four 60-s verbal fluency tasks: for letter-cued trials, they were asked to report as many words as possible beginning with the letters “s” and “p.” For the category-cued trials they were asked to report as many animals and supermarket items as possible. Both were drawn from the Calibrated Ideational Fluency Assessment (CIFA; Schretlen and Vannorsdall, 2010). Responses were recorded using a studio-quality microphone and Audacity (version 1.2.6) software. Verbal fluency productions were transcribed and scored offline.

Verbal fluency protocols were scored following the Hopkins qualitative verbal fluency system (Ledoux et al., 2009), which is a modification of the criteria developed by Troyer et al. (1997). The system uses specified criteria to determine the total number of acceptable words generated, numbers of switches and clusters, mean cluster size, and percent words in clusters for both letter-cued and category-cued verbal fluency tasks. All scoring was conducted by trained research assistants who were blind to the participant stimulation condition.

### Analyses

In the full sample (*n* = 40), multivariate ANCOVAs adjusting for participant age and education were used to test for differences in fluency output by condition (anodal vs. cathodal) and to compare active (anodal or cathodal) vs. sham stimulation. We also assessed difference scores in verbal fluency productions during anodal and sham stimulation (i.e., anodal minus sham) relative to the difference scores during cathodal and sham stimulation (i.e., cathodal minus sham).

After equating the groups for age and education, our sample size (*n* = 24) was not suitable for a multivariate ANCOVA. We therefore examined the distribution of each dependent variable and conducted between-groups comparisons of verbal fluency output by the anodal and cathodal stimulation groups using independent samples *t*-tests (for normally distributed variables) and the Wilcoxon signed-ranks test (for variables with non-normal distributions). Within-groups analyses were used to compare fluency during active (anodal or cathodal) vs. sham stimulation using paired *t*-tests. Independent samples *t*-tests or Wilcoxon signed-ranks tests were also used to assess difference scores in verbal fluency productions during anodal and sham stimulation (i.e., anodal minus sham) relative to the difference scores during cathodal and sham stimulation (i.e., cathodal minus sham).

## Results

For the sample as a whole, multivariate ANCOVAs revealed no significant within- or between-groups effects of tDCS on any of the verbal fluency variables with respect to either letter- or category-cued fluency tasks (*p*s ≥ 0.25).

In the subsample of participants in which groups were well matched with respect to potential confounders, there were no significant effects of tDCS on overall letter-cued fluency productivity (*p*s > 0.05). Similarly, for letter-cued fluency there were no significant between-groups (anodal vs. cathodal) or within-groups (active vs. sham) effects of stimulation on any of the qualitative fluency measures (*p*s > 0.05).

With respect to possible effects of anodal and cathodal stimulation on category-cued verbal fluency, the overall productivity of the two groups did not differ significantly and there were no differences in qualitative aspects of verbal fluency between the groups (*p* > 0.05).

When we examined the distributions of dependent variables, two (percent words in clusters during active anodal stimulation and percent words in clusters during sham anodal stimulation) violated the assumption of normality, whereas the others did not. Analyses revealed a trend toward greater category-cued verbal fluency productivity during active anodal relative to sham stimulation [active *M* = 25.9, SD = 6.2; sham *M* = 23.0, SD = 5.6; *t*(11) = 2.14, *p* = 0.055]. Active anodal stimulation was also associated with the production of more words in clusters relative to sham stimulation [active *M* = 22.1, SD = 7.5; sham *M* = 18.3, SD = 8.1; *t*(11) = 2.41, *p* = 0.035]. During active anodal tDCS, participants also showed a trend toward reporting a greater percentage of words in clusters relative to sham tDCS (active Median = 87.3, Interquartile range = 15.1; sham Median = 78.4, Interquartile range = 14.9; *Z* = −1.88; *p* = 0.06). No differences between active and sham tDCS were found for the number of switches or mean cluster size (*p*s > 0.05).

**Figure 1:**
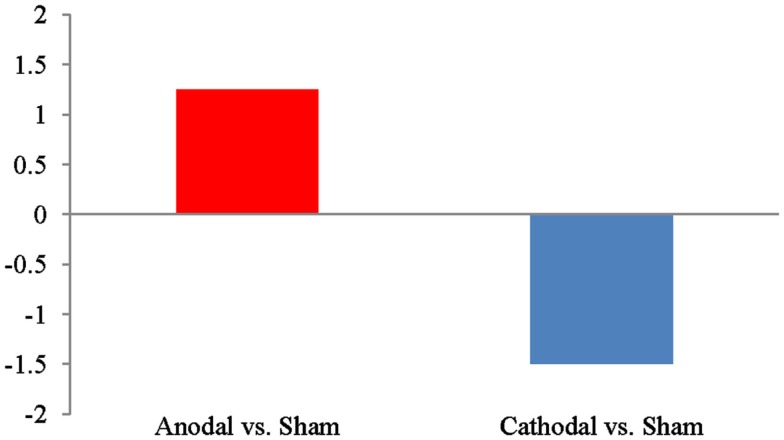
**Effects of active stimulation conditions compared to sham stimulation with respect to the numbers of words in clusters during a category-cued fluency task**.

**Figure 2:**
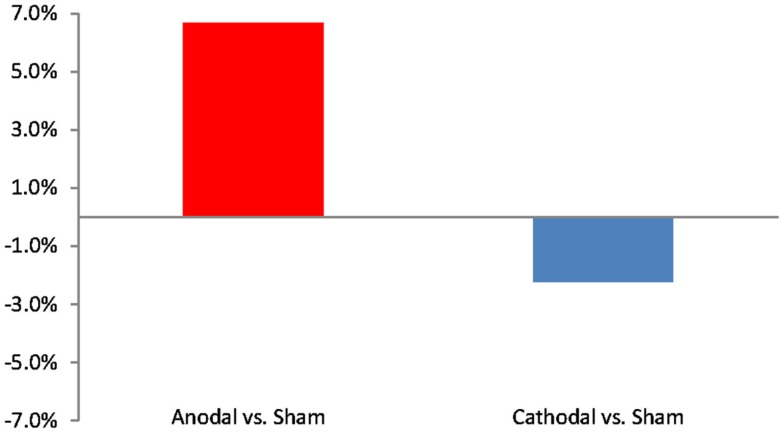
**Effects of active stimulation conditions compared to sham stimulation with respect to the percent words in clusters during a category-cued fluency task**.

We next compared differences in the number of word clusters participants produced during active than sham stimulation as a function of current polarity. Compared to sham stimulation, participants showed a net increase in word clusters during active anodal stimulation (*M* = 1.3, SD = 2.5), whereas they showed a net decrease in word clusters during active cathodal stimulation (*M* = −1.5, SD = 3.6). This difference was significant, *t*(22) = −2.21; *p* = 0.038 and is depicted in Figure 1. Similarly, compared to sham stimulation, active anodal tDCS led to a 6.6% increase in the percent of words in clusters, whereas active cathodal stimulation produced a 2.2% reduction in the percent words in clusters. This difference [*t*(22) = −2.12, *p* = 0.046] is shown in Figure 2. There were no significant effects of tDCS on switching or mean cluster size (*p*s > 0.05).

## Discussion

We hypothesized that active anodal and cathodal tDCS would, respectively, enhance and diminish overall productivity on tests of verbal fluency. Based on our placement of the active electrode over the left dorsolateral prefrontal cortex, we expected to find more prominent tDCS effects on letter- than category-cued tasks and on measures of controlled (i.e., switching) than automatic (i.e., clustering) word retrieval processes. In a subsample of participants matched for basic demographic characteristics, our results provide partial support for our hypotheses in that anodal tDCS selectively enhanced aspects of verbal fluency while cathodal stimulation inhibited the same processes. However, our predictions regarding the type of fluency task and the qualitative aspects of fluency performance that would be most affected by tDCS were not supported.

In fact, we found that active anodal tDCS affected category-cued fluency productivity but had no discernible effects on letter-cued verbal fluency. Nor did tDCS alter controlled cognitive aspects of word retrieval (i.e., switching), despite our application of stimulation over the left dorsolateral prefrontal cortex, an area often associated with executive functioning and set-shifting. Rather, we found a nearly three-word increase in productivity on category-cued verbal fluency tasks in the anodal stimulation condition relative to the sham condition. Analyses of the qualitative aspects of verbal fluency productions suggest that this enhanced productivity was likely due to the increased clustering seen with anodal stimulation relative to both the sham and cathodal stimulation, and not due to changes in switching. Although more modest in its effect, cathodal stimulation also reduced clustering relative to sham stimulation.

The fact that we found effects of tDCS exclusively for category fluency, and not letter fluency, differs from the two other studies of tDCS and verbal fluency in healthy adults. Iyer et al. (2005) found facilitative effects of 2 mA of anodal tDCS on overall productivity on a test of letter-cued fluency compared to sham and cathodal conditions. However, they did not find effects of tDCS in their initial experiment which used a lower current intensity (1 mA) and participants in their study did not complete category-cued fluency tasks. Cattaneo et al. (2011) also used 2 mA of anodal stimulation and found improved productivity for both letter and category-cued fluency relative to sham stimulation. Although group means are not presented, a figural representation of the data suggests a larger magnitude of effect for category-cued relative to letter-cued fluency. Thus, our lack of findings for letter-cued fluency may be due to our decision to use 1 mA rather than 2 mA of current. We chose to use 1 mA because pilot testing revealed that subjects could reliably detect active stimulation at 2 mA whereas they could not at 1 mA. Thus, in our effort to blind our study participants to the stimulation condition, we may have also reduced the effectiveness of the experimental intervention. In addition, we administered sham and active tDCS separated by a 90-min washout during each session. We based this decision on evidence that the cortical excitability effects of short duration tDCS typically return to baseline by 60–90 min after the cessation of stimulation (Nitsche and Paulus, 2001). However, if active tDCS stimulation combined with directed cognitive activity produces longer-last effects, this could have limited our ability to detect the behavioral effects of active tDCS. Future studies should explore whether increasing administration of a greater current density would produce effects for letter-cued fluency, as well as whether administering stimulation to more posterior regions would produce effects on both types of word fluency tasks.

Another weakness of this study is the heterogeneity of the initial study sample and unbalanced randomization into study groups. Participants were recruited through two methods, flyers placed on the Johns Hopkins University and medical campuses and through Internet ads (i.e., Craigslist). As a result, we recruited a rather homogenous group of young, well-educated participants along with a larger group of individuals having more diverse demographic characteristics. When examining the effects of tDCS on verbal fluency, we found no effects of tDCS within the full sample of participants. One hypothesis for this lack of findings is that the healthy, young, highly educated individuals who were over-represented in the anodal condition were already performing at ceiling and masked the effects of tDCS within the remaining participants. In fact, when the sample was trimmed to form two groups matched for relevant characteristics only then were the effects of tDCS apparent. Future studies should further explore the role of patient characteristics in relation to participant responsiveness to experimental interventions.

A related limitation to the current study is that, due to the small size of the final sample, we were unable to use multivariate ANOVA. Nor did we adjust for multiple comparisons. The latter decision was based on the fact that this was an exploratory study that aimed to determine whether tDCS could selectively alter controlled and automatic aspects of verbal fluency productions in healthy adults. We believe that the present findings, while relatively weak, suggest that tDCS can alter these word retrieval processes, as well as overall productivity on such tasks.

A final weakness is that this study employed a single-blind rather than double-blind experimental design. The tDCS device we used is not programmable in a way that permits one to blind both the experimenter and participant to the experimental condition. We did have one experimenter administer the cognitive testing while another operated the tDCS device, but this did not blind the machine operator to the experimental condition, and the other experimenter usually could discern whether a recipient was receiving active or sham stimulation. A procedural “workaround” for this limitation is possible but cumbersome in practice, and was not used in the present study. Fully programmable tDCS devices that overcome this limitation are recommended for use in future studies.

To our knowledge, this is the first study to investigate the utility of tDCS as a means of altering automatic and controlled aspects of speeded lexical during both letter and category word fluency tasks in neurologically healthy adults. We found that anodal tDCS was associated with an increase in overall productivity during a category-guided verbal fluency task, and that anodal stimulation led to a relative increase in clustering whereas cathodal stimulation had the opposite effect. These findings, although preliminary, suggest that tDCS may be an effective tool in ameliorating language dysfunction in disorders characterized by deficient activation or functioning of the semantic network. Our ongoing work is exploring this issue in such individuals including those with aphasia, autism spectrum disorders, and schizophrenia.

## Conflict of Interest Statement

Under an agreement with Psychological Assessment Resources, Inc., Drs. Tracy D. Vannorsdall and David J. Schretlen are entitled to a share of royalties on sales of the CIFA. The terms of these arrangements are being managed by the Johns Hopkins University in accordance with its conflict of interest policies. None of the other authors report any financial interests or potential conflicts of interests.
